# Reject Rates of Radiographic Images in Dentomaxillofacial Radiology: A Literature Review

**DOI:** 10.3390/ijerph18158076

**Published:** 2021-07-30

**Authors:** Andy Wai Kan Yeung, Natalie Sui Miu Wong

**Affiliations:** 1 Oral and Maxillofacial Radiology, Applied Oral Sciences and Community Dental Care, Faculty of Dentistry, The University of Hong Kong, Hong Kong, China; 2 Oral and Maxillofacial Surgery, Faculty of Dentistry, The University of Hong Kong, Hong Kong, China; smwong26@hku.hk

**Keywords:** cone beam computed tomography, dental imaging, image retake, bitewing, periapical, panoramic, lateral cephalography

## Abstract

This report surveyed the image reject rates of intra-oral, extra-oral, and cone-beam computed tomography (CBCT) imaging in the academic literature. PubMed, Web of Science, and Scopus databases were queried in mid-April 2021. Manual screening of the reference lists of the identified publications was performed to identify papers missed from the database search. All publications returned by the searches were initially included. Exclusion criteria included irrelevance, no reporting of reject rate, no access to the article, and not original article. The total number of images and the number of rejects were recorded for each type of radiographic images. Factors and commonest errors associated with the rejects were recorded. Twenty-six original articles were identified and reviewed. The average reject rate was 11.25% for bitewings, 16.38% for periapicals, 4.10% for panoramics, 6.08% for lateral cephalography, and 2.77% for CBCT. Positioning error and patient movement were two common reasons for the rejects. The average reject rates computed from data pooled across studies should form the reference values for quality assurance programs to follow. Future reject analysis studies should report more radiographic parameters such as type of collimation for intra-oral radiography and patient posture for CBCT.

## 1. Introduction

Dentomaxillofacial radiology (DMFR) is an essential part of dentistry as the visualization of the internal structures often cannot be achieved by clinical examination yet is crucial for diagnosis and treatment planning [1,2]. To balance the risks and benefits of acquiring radiographic images, radiation dose protection principles of justification, optimization, and dose limitation should be observed by considering the notions of ALARA (As Low As Reasonably Achievable), ALADA (As Low As Diagnostically Acceptable), or, more recently, ALARAIP (As Low As Diagnostically Acceptable being Indication-oriented and Patient-specific) [3,4,5,6].

One way to protect the patients is to reduce the radiation dose by various means. For intra-oral radiography, the use of rectangular collimation was found to significantly reduce the radiation dose compared to round collimation [7]. For cone-beam computed tomography (CBCT), the use of low dose protocols for suitable indications might effectively reduce the radiation dose without undermining the diagnostic quality of the images [8]. The other way to protect the patients is to ensure that diagnostically acceptable radiographic images are obtained in the first attempt without the need of a retake. For example, film holders should be used for bitewing and periapical radiographs to align the image receptor precisely with the X-ray beam [9]. Besides, adequate formal training for taking dental radiographs could reduce the frequencies of radiographic errors [10]. Meanwhile, various head stabilizers could be deployed during a CBCT scan to reduce patient head movement [11]. It remains unclear whether the reject rates of dental radiographic imaging were comparable around the world. To the best of the authors’ knowledge, there was no report that summarized the data in the existing literature. Such information regarding the image reject rates in the dentomaxillofacial radiology literature provides reference levels for quality assurance protocols, and is beneficial to identify potential pitfalls in the practices. 

The purposes of the present literature review were to unveil the reject rates of dental radiographic images reported in the existing academic literature, and to identify the factors and commonest errors associated with the rejects.

## 2. Materials and Methods

On 16 April 2021, three literature databases, namely PubMed, Web of Science, and Scopus, were queried. The same search string was applied to all three databases as: (reject* OR retake* OR reexpos*) AND (analy* OR rate*) AND (dental OR oral OR maxillofacial) AND (radio*). Title and abstract fields were searched for PubMed, whereas title, abstract, and keyword fields were searched for Web of Science and Scopus. All publications returned by the searches were initially included. Exclusion criteria included irrelevance, no reporting of reject rate, no access to the article, and not original article. No additional filter was placed to limit the original study design (e.g., prospective or retrospective), publication year, and language.

**Figure 1:**
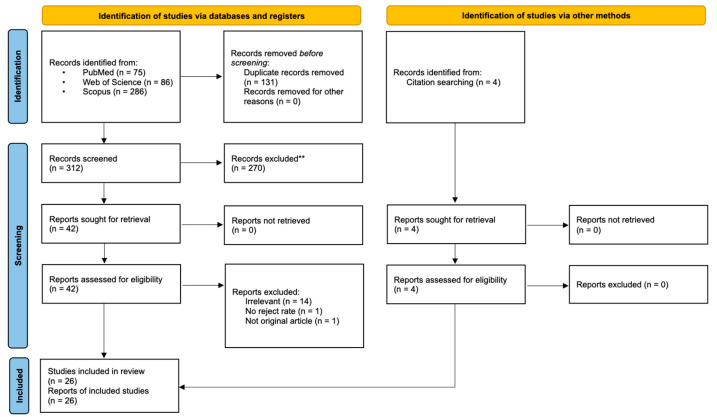
A flow chart showing the screening process of the literature search. The flow chart was adapted from Preferred Reporting Items for Systematic reviews and Meta-Analyses (PRISMA) 2020 version [12].

The search initially returned with 447 publications. After removing duplicates manually in Excel (Version 16.50 for Mac, Microsoft, Washington, DC, USA), 312 publications remained. After screening and excluding unsuitable publications with specific reasons, 22 studies remained. Reference search added another four studies. In total, 26 studies entered the review (Figure 1). Each author did the screening independently and a final consensus was reached. The following items of information were recorded from each study included in the review: number of radiographs taken, number of radiographs rejected, operator type, patient age, factors that affected the reject rate, and commonest errors that accounted for >5% of the rejects. For plain radiographic studies, the receptor type and film-focus distance (FFD) were recorded. In addition, the collimator (cone) type was noticed. For CBCT studies, the scan posture, head stabilizer, and scan time were recorded.

Ethical approval was not applicable to this review. The protocol of this review was not preregistered in online databases such as PROSPERO.

## 3. Results

Twenty-six included articles are listed in Table 1, Table 2 and Table 3 [13,14,15,16,17,18,19,20,21,22,23,24,25,26,27,28,29,30,31,32,33,34,35,36,37,38]. Reject rate of intra-oral radiography was reported in 17 articles (Table 1), extra-oral radiography in 7 articles (Table 2), and CBCT in 5 articles (Table 3).

### 3.1. Intra-Oral Radiography

In intra-oral radiography, studies together reported a mean bitewing reject rate of 11.25% (1663/14,779) with a range of 1.95–27.73%. For periapicals, the mean reject rate was 16.38% (2827/17,263) with a range of 2.58–34.42%. For studies reporting data from bitewings and periapicals altogether, the mean reject rate was 6.69% (3910/58,407) with a range of 2.96–39.02%. Studies published since 2017 reported data from digital imaging only. Seven studies used rectangular collimation, and eight studies did not report details on collimation. The studies were largely heterogeneous in terms of the operator, patient age, and factors associated with reject rate. Overall, it seemed that the reject rate was lower for film systems than digital systems. One explanation could be that intra-oral (IO) sensors are bulkier than films, making them less tolerable by the patients and more difficult to be positioned ideally. For digital systems, photostimulable phosphor plates had a lower reject rate than intra-oral sensor. Anatomically, the mandible had a lower reject rate than maxilla, and similarly the anterior region than the posterior region. Operator type and related work experience also influenced the reject rate. Not surprisingly, the commonest errors leading to reject were positioning error and cone cut. Only two studies described specific indications for the imaging, e.g., one study evaluated periapicals taken for endodontic treatment [18], and one study evaluated periapicals taken for lower third molars [25].

### 3.2. Extra-Oral Radiography

Panoramic radiography had a mean reject rate of 4.10% (482/11,753) with a range of 2.89–11.65%. Lateral cephalography had a higher mean reject rate of 6.08% (74/1218), based on two studies that reported a rate of 4.19% and 13.75%, respectively. One study reported an overall reject rate of 5.86% (137/2339) for all extra-oral radiography. Three studies used film systems, two used digital systems, and two used both. Operator type and patient age varied between studies. Positioning error and patient movement were mentioned in at least two studies as the commonest reasons for the rejects. Only two studies described specific indications for the imaging, e.g., one study evaluated lateral cephalograms taken for orthodontic treatment [29], and one study evaluated panoramics taken for lower third molars [15].

### 3.3. CBCT

Interestingly, the mean CBCT reject rate was only 2.77% (223/8060) and was lower than that of intra-oral and extra-oral imaging. The range was 1.64–20.25% based on five studies. The details of image acquisition were largely unreported, such as patient posture, head stabilizer, scan time, and operator type. Four studies involved CBCT scans from both pediatric and adult patients, whereas the remaining study evaluated scans from pediatric patients below 12 years of age. Inadequate field of view and patient movement were again the recurring reasons for the rejects.

## 4. Discussion

The present literature review provided an overview of the reject rates of and reasons for intra-oral, extra-oral, and CBCT imaging published in the academic literature. According to the results, the investigations have covered CBCT imaging since 2015. This seemed to be late, as the first CBCT devices were introduced in 1998–1999 [39,40] and the first systematic review that evaluated diagnostic ability of CBCT was published in 2008 [41]. Reject analysis can identify recurring problems leading to unacceptable diagnostic value of radiographic images and devise person- or institution-specific remedies accordingly [42]. Therefore, its conductance and publication should be encouraged, so that the international DMFR community could establish recommended reference levels/values for quality assurance programs to follow.

For intra-oral radiography studies, the type of collimation was often not reported. Previously it was reported that the use of a rectangular collimator could reduce 40–92% of radiation dose compared to a circular collimator [7], and the use of the former did not significantly affect the diagnostic yield of bitewings [31]. One could argue that circular collimation should have a smaller risk of rejects due to cone cut, and thus future studies should consider reporting the type of collimation to allow a better comparison of data obtained by each collimation. Meanwhile, many of the studies reviewed here reported the reject rate based on bitewing and periapical imaging combined, e.g., from a full mouth series that consisted multiple periapicals and a pair (or two pairs) of bitewings. The pooled data showed that the reject rate of periapicals was 5% higher than that of bitewings, and therefore future studies should consider reporting statistics separately for each type of images. One explanation for the higher reject rate of periapicals is that periapical radiography requires the insertion of the image receptor deep into the oral cavity. In order to capture the root apices, the receptor most likely will touch the palatal vault or the floor of the mouth, causing discomfort and gagging. Meanwhile, bitewing radiography covers the tooth crowns only and hence the receptor usually is more tolerated by the patient. The studies reviewed suggested that the reject rate was higher for digital imaging than film, and particularly higher for intra-oral sensor than photostimulable phosphor plate. The underlying reasons were not investigated in depth, but several explanations were proposed, such as convenience of instant chairside display by intra-oral sensor [13], and inability to position mesially enough in the premolar region due to the bulkiness of the sensor [43]. Operator types varied from dental students, dental hygienist students, to dentists and radiographers. With such limited data, it remained to be elucidated if their reject rates were significantly different. Students could be assumed to have a higher rate as work experience was sometimes reported by the reviewed studies as an associated factor. For quality assurance purpose, perhaps different reject rate targets should be set for each imaging type and operator type (and even for patient age, child vs. adult) eventually. With the limited data available in the existing literature, the overall reject rate for intra-oral radiography stood at approximately 9% and this should be a reference value for institutions to consider achieving.

For extra-oral radiography studies, data from panoramic imaging was reported by five studies whereas lateral cephalography by two studies. The overall reject rates were 4% and 6%, respectively. More reject analysis on cephalography should be encouraged in the future, and that future studies should report more imaging parameters. For instance, lateral cephalography could be acquired by either one-shot or continuous scanning mode, depending on the radiographic device. It would be reasonable to deduce that one-shot mode, with a shorter exposure time, should be less affected than continuous scanning by patient movement, a factor leading to frequent rejects reported in one study [28].

For CBCT studies, the overall reject rate was approximately 3%, considerably lower than the values from intra-oral and extra-oral imaging. One potential reason could be that CBCT imaging had higher radiation dose than plain radiography, so operators tended to avoid rejects and thus retakes. Meanwhile, patient posture was reported in one study only. The effect of patient posture (sitting, standing, or supine) on reject rates was never investigated. Its effect on patient movement was similarly rarely investigated, if not none [44]. A simulation study [45] and a retrospective image inspection study [46] showed that supine position seemed to produce less motion artifact. A patient study on children similarly reported that supine position was associated with reduced severe head motion [47]. Therefore, future reject analyses should report patient posture. Stabilizer type, scan time, and operator type were also frequently missed by the studies reviewed here, and these factors should be crucial to patient movement too. In contrast to intra-oral radiography, extra-oral and CBCT imaging often take approximately 10 s up to nearly a minute, and therefore patient movement can only be reduced but not totally eliminated. In patients under 18 years of age, reject rates were highest for scans of dento-alveolar trauma (50%), surgical application (36%), and generalized evaluation of developing dentition (33%) [36]. Meanwhile, for CBCT rejects due to incomplete FOV coverage, the most frequently involved reasons were incomplete coverage of the apex of impacted lower third molars and/or pericoronal pathology (22.4%), and incomplete coverage of the opposite teeth of implant planning sites, associated maxillary sinus floor, and/or sleeves of radiographic stent (20.4%) [23]. More data should be reported by future studies regarding the regions of interest to be observed by the rejected images.

One limitation of this review was that many studies did not report common reasons for the rejects, but common errors instead. Whilst common errors could be of interest, errors do not necessarily lead to rejects. Besides, publications not indexed by the major databases and unpublished data were potentially missed from the current review.

## 5. Conclusions

It was found that the average reject rate was 11.25% for bitewings, 16.38% for periapicals, 4.10% for panoramics, 6.08% for lateral cephalography, and 2.77% for CBCT. Positioning error and patient movement were two common reasons for the rejects of intra-oral and extra-oral imaging, respectively. Efforts should be made to modify existing radiographic workflow and evaluate the effectiveness of the modifications. For instance, perhaps film holders could be fabricated with soft yet autoclavable materials. The infection control barrier of the image receptor should have no sharp corners or wrapped by a piece of gauze to reduce discomfort upon pressing against the palate or floor of mouth. As patient movement during extra-oral radiography is unavoidable, efforts should be made to minimize it and manufacturers should upgrade the image reconstruction algorithms to further correct for motion artifact.

Some studies reported that additional teaching and increased working experience could reduce the reject rates, and generally staff had a lower reject rate than students. These findings suggested that more regular radiographic training should be recommended for all personnel working in dental radiography focusing on techniques for correct positioning and patient movement reduction.

## Figures and Tables

**Table 1:** Reject rates reported for intra-oral radiography.

Study	Modality	Reject N	Total N	Reject %	Receptor Type	FFD (cm)	Collimator	Operator	Patient Age	Factors Associated with Reject Rate (A < B Means B Had Higher Reject Rate)	Common Errors Leading to Reject (Account for >5%)
Jensen (1978) [24]	FMX (14 Peri + 4 BW)	300	5076	5.91	Film	40	Circular	Dental undergrads	Adults	Additional teaching; Ant < Post; work experience	Positioning
Gratt (1985) [19]	FMX (21)	130	1220	10.66	Film; xeroradiography	43	N.A.	N.A.	>21 y	Film < xeroradiography	N.A.
Gound (1994) [18]	Peri (for endo)	92	402	22.89	Film	N.A.	N.A.	Dental undergrads	Adults	Md < Mx; EndoRay < hemostat	N.A.
Nixon (1995) [28]	BW	39	1999	1.95	Film	N.A.	N.A.	Multiple levels	All age	Radiographer < undergrad & dentist < radiographer student & postgrad	Positioning
	Peri	163	6313	2.58							
Szymkowiak (1995) [35]	Peri + BW	119	305	39.02	Film	N.A.	N.A.	Dentists	N.A.	N.A.	Positioning; horizontal angulation; cone cut; vertical angulation; density and contrast
Versteeg (1998) [37]	Peri	34	100	34.00	Film; IO sensor	N.A.	Rectangular	Radiographers	N.A.	Film < IO sensor	N.A.
Sommers (2002) [33]	FMX (14 Peri + 4 BW)	371	1008	36.81	Film; IO sensor	N.A.	N.A.	DH students	Manikin	Film < IO sensor	N.A.
Hellen-Halme (2004) [22]	Peri + BW	878	4657	18.85	Film (mainly); digital	N.A.	N.A.	Dentists	>65 y	Film < digital	Positioning
Chau (2006) [16]	Peri + BW	418	2334	17.91	Film	N.A.	Rectangular	DH students	>8 y	Laser guided collimator < standard; work experience	N.A.
Mupparapu (2007) [26]	Peri + BW	1017	34312	2.96	Film	N.A.	Rectangular	Dental undergrads; dental surgery assistants	N.A.	Staff < student	N.A.
Matzen (2009) [25]	Peri (of lower 8 s)	98	298	32.89	IO sensor; PSP	N.A.	Rectangular	N.A.	>18 y	Women < men; discomfort; X-ray system	Positioning
Parrott (2011) [31]	BW	374	3000	12.47	Film	N.A.	Both	N.A.	Adult	N.A.	Vertical distortion; horizontal angulation
Acharya (2015) [13]	Peri + BW	677	9495	7.13	Film	N.A.	Rectangular	Multiple levels	N.A.	Md < Mx	N.A.
Nenad (2016) [27]	BW	183	660	27.73	N.A.	N.A.	N.A.	DH students	N.A.	Work experience	N.A.
Pacheco-Pereira (2017) [30]	BW	90	1296	6.94	IO sensor; PSP	N.A.	Rectangular	DH students	All age	PSP < IO sensor	Positioning; cone cut; patient not biting
	Peri	33	590	5.59							
Yusof (2017) [38]	BW	345	2284	15.11	IO sensor	N.A.	N.A.	Dental undergrads	N.A.	N.A.	Positioning
	Peri	1978	5746	34.42							
Senior (2018) [32]	BW	632	5540	11.41	IO sensor; PSP	40	Rectangular	Dental undergrads	All age	PSP < IO sensor	Positioning; cone cut; horizontal angulation
	Peri	429	3814	11.25							

BW, bitewing. DH students, dental hygienist students. FFD, focal-film distance. FMX, full mouth series. IO sensor, intra-oral sensor. Md, mandible. Mx, maxilla. Peri, periapical. PSP, photostimulable phosphor plates.

**Table 2:** Reject rates reported for extra-oral radiography.

Study	Modality	Reject N	Total N	Reject %	Receptor Type	Operator	Patient Age	Factors Associated with Reject Rate (A < B Means B Had Higher Reject Rate)	Common Errors Leading to Reject (Account for >5%)
Ortendahl (1994) [29]	Lat ceph (for orthodontics)	33	240	13.75	Film	N.A.	N.A.	N.A.	N.A.
Nixon (1995) [28]	Pan	212	6395	3.32	Film	Multiple levels	All age	N.A.	Positioning; patient movement
	Lat ceph	41	978	4.19					
Benediktsdottir (2003) [15]	Pan (for lower 8 s)	38	497	7.65	Digital	Radiographers	18–44 y	X-ray system	N.A.
Hellen-Halme (2004) [22]	Pan	24	206	11.65	Film; digital	Dentists	>65 y	N.A.	Unsharpness; contrast too low
Ekstromer (2014) [17]	Pan	55	1904	2.89	Film; digital	N.A.	All age	N.A.	N.A.
Acharya (2015) [13]	All EO	137	2339	5.86	Film	Multiple levels	N.A.	Radiographers < Dental postgrads	Positioning; improper exposure; patient movement; improper bite; film fog
Behroozi (2015) [14]	Pan	153	2751	5.56	Digital	N.A.	N.A.	N.A.	Positioning; not sticking the tongue to the hard palate

EO, extra-oral. Lat ceph, lateral cephalography. Pan, panoramic.

**Table 3:** Reject rates reported for CBCT.

Study	Reject N	Total N	Reject %	Patient Posture	Stabilizer	Scan Time	Operator	Patient Age	Factors Associated with Reject Rate (A < B Means B Had Higher Reject Rate)	Common Errors Leading to Reject (Account for >5%)
Spin-Neto (2015) [34]	16	248	6.45	Seated	Chin rest; head clamp	17–22 s	N.A.	All age	Small FOV < large FOV	Inadequate FOV
Greenall (2016) [20]	29	1010	2.87	N.A.	Chin rest; head strap	18–40 s	Radiographers	5–80 y	X-ray system; Md < Mx	Patient movement; inadequate FOV
Habibi (2019) [21]	82	4986	1.64	N.A.	N.A.	N.A.	N.A.	All age	Large FOV < small FOV	Patient movement
Van Acker (2019) [36]	16	79	20.25	N.A.	N.A.	N.A.	N.A.	<12 y	N.A.	N.A.
Hung (2020) [23]	80	1737	4.61	N.A.	N.A.	N.A.	Dentists	All age	X-ray system; Adult < under 12 y	Patient movement; inadequate FOV

FOV, field of view. Md, mandible. Mx, maxilla.

## References

[1] Boeddinghaus R., Whyte A. (2018). Trends in maxillofacial imaging. Clin. Radiol..

[2] Yeung A.W.K., Wong N.S.M. (2021). Medial Sigmoid Depression of the Mandibular Ramus as a Lesion-Mimicking Anatomical Variation: A Systematic Review. Int. J. Environ. Res. Public Health.

[3] Jaju P.P., Jaju S.P. (2015). Cone-beam computed tomography: Time to move from ALARA to ALADA. Imaging Sci. Dent..

[4] International Commission on Radiological Protection (2007). The 2007 Recommendations of the International Commission on Radiological Protection. ICRP publication 103. Ann. Icrp.

[5] Oenning A.C., Jacobs R., Pauwels R., Stratis A., Hedesiu M., Salmon B., Group D.R. (2018). Cone-beam CT in paediatric dentistry: DIMITRA project position statement. Pediatr. Radiol..

[6] Yeung A.W.K. (2019). The “As Low As Reasonably Achievable” (ALARA) principle: A brief historical overview and a bibliometric analysis of the most cited publications. Radioprotection.

[7] Shetty A., Almeida F.T., Ganatra S., Senior A., Pacheco-Pereira C. (2019). Evidence on radiation dose reduction using rectangular collimation: A systematic review. Int. Dent. J..

[8] Yeung A.W.K., Jacobs R., Bornstein M.M. (2019). Novel low-dose protocols using cone beam computed tomography in dental medicine: A review focusing on indications, limitations, and future possibilities. Clin. Oral Investig..

[9] American Dental Association Council on Scientific Affairs (2006). The use of dental radiographs: Update and recommendations. J. Am. Dent. Assoc..

[10] Bissoon A., QWhaites E., Moze K., Naidu R. (2012). Evaluation of common operator errors in panoramic radiography in Trinidad and Tobago: A comparison of formally vs informally trained operators. West. Indian Med. J..

[11] Macleod I., Heath N. (2008). Cone-beam computed tomography (CBCT) in dental practice. Dent. Update.

[12] Page M.J., McKenzie J.E., Bossuyt P.M., Boutron I., Hoffmann T.C., Mulrow C.D., Shamseer L., Tetzlaff J.M., Akl E.A., Brennan S.E. (2021). The PRISMA 2020 statement: An updated guideline for reporting systematic reviews. BMJ.

[13] Acharya S., Pai K.M., Acharya S. (2015). Repeat film analysis and its implications for quality assurance in dental radiology: An institutional case study. Contemp. Clin. Dent..

[14] Behroozi H., Afkandeh R. (2015). Causes of Repeating Digital Panoramic Radiographs in Maxillofacial Imaging Centers. J. Maz. Univ. Med. Sci..

[15] Benediktsdottir I., Hintze H., Petersen J., Wenzel A. (2003). Image quality of two solid-state and three photostimulable phosphor plate digital panoramic systems, and treatment planning of mandibular third molar removal. Dentomaxillofac. Radiol..

[16] Chau A., Li T., Wong J. (2006). A randomized double blinded study to assess the efficacy of a laser-guided collimator on dental radiography training. Dentomaxillofac. Radiol..

[17] Ekstromer K., Hjalmarsson L. (2014). Positioning errors in panoramic images in general dentistry in Sörmland County, Sweden. Swed. Dent. J..

[18] Gound T.G., DuBois L., Biggs S.G. (1994). Factors that affect the rate of retakes for endodontic treatment radiographs. Oral Surg. Oral Med. Oral Pathol..

[19] Gratt B.M., Sickles E.A., Littman R.I. (1985). Comparison of dental xeroradiography and conventional film techniques for the frequency and significance of image artifacts. Oral Surg. Oral Med. Oral Pathol..

[20] Greenall C., Thomas B., Drage N., Brown J. (2016). An audit of image quality of three dental cone beam computed tomography units. Radiography.

[21] Habibi Y., Habibi E., Al-Nawas B. (2019). Re-exposure in cone beam CT of the dentomaxillofacial region: A retrospective study. Dentomaxillofac. Radiol..

[22] Hellén-Halme K., Johansson P.-M., Håkansson J., Petersson A. (2004). Image quality of digital and film radiographs in applications sent to the Dental Insurance Office in Sweden for treatment approval. Swed. Dent. J..

[23] Hung K., Hui L., Yeung A.W.K., Scarfe W.C., Bornstein M.M. (2020). Image retake rates of cone beam computed tomography in a dental institution. Clin. Oral Investig..

[24] Jensen T.W. (1978). Improved reliability of dental radiography by application of X-ray beam-guiding instruments: A two-year report. J. Dent. Educ..

[25] Matzen L.H., Christensen J., Wenzel A. (2009). Patient discomfort and retakes in periapical examination of mandibular third molars using digital receptors and film. Oral Surg. Oral Med. Oral Pathol. Oral Radiol. Endodontol..

[26] Mupparapu M., Jariwala S., Singer S., Kim I., Janal M. (2007). Comparison of re-exposure rates of intraoral radiographs between dental students and trained dental assistants in an oral and maxillofacial radiology clinic. Dentomaxillofac. Radiol..

[27] Nenad M.W., Halupa C., Spolarich A.E., Gurenlian J.R. (2016). A dental radiography checklist as a tool for quality improvement. J. Dent. Hyg..

[28] Nixon P., Thorogood J., Holloway J., Smith N. (1995). An audit of film reject and repeat rates in a department of dental radiology. Br. J. Radiol..

[29] Örtendahl T., Borrman H., Gröndahl H. (1994). Quality assessment of lateral cephalograms amongst radiologists and orthodontists. Br. J. Orthod..

[30] Pachêco-Pereira C., Brandelli J., Senior A. (2017). Re-exposure rates of digital intraoral images taken by undergraduate dental hygiene students. Can. J. Dent. Hyg..

[31] Parrott L., Ng S. (2011). A comparison between bitewing radiographs taken with rectangular and circular collimators in UK military dental practices: A retrospective study. Dentomaxillofac. Radiol..

[32] Senior A., Winand C., Ganatra S., Lai H., Alsulfyani N., Pachêco-Pereira C. (2018). Digital Intraoral Imaging Re-Exposure Rates of Dental Students. J. Dent. Educ..

[33] Sommers T.M., Mauriello S.M., Ludlow J.B., Platin E., Tyndall D.A. (2002). Pre-clinical performance comparing intraoral film and CCD-based systems. J. Dent. Hyg..

[34] Spin-Neto R., Matzen L.H., Schropp L., Gotfredsen E., Wenzel A. (2015). Factors affecting patient movement and re-exposure in cone beam computed tomography examination. Oral Surg. Oral Med. Oral Pathol. Oral Radiol..

[35] Szymkowiak L., Sarll D., Horner K. (1995). Some factors affecting the standards of radiography in general dental practice. Br. Dent. J..

[36] Van Acker J.W., Jacquet W., Dierens M., Martens L.C. (2019). A reject analysis of cone-beam CTs in under-aged patients. Dentomaxillofac. Radiol..

[37] Versteeg C., Sanderink G., Van Ginkel F., Van der Stelt P. (1998). An evaluation of periapical radiography with a charge-coupled device. Dentomaxillofac. Radiol..

[38] Yusof M.Y.P.M., Rahman N.L.A., Asri A.A.A., Othman N.I., Mokhtar I.W. (2017). Repeat analysis of intraoral digital imaging performed by undergraduate students using a complementary metal oxide semiconductor sensor: An institutional case study. Imaging Sci. Dent..

[39] Arai Y., Tammisalo E., Iwai K., Hashimoto K., Shinoda K. (1999). Development of a compact computed tomographic apparatus for dental use. Dentomaxillofac. Radiol..

[40] Mozzo P., Procacci C., Tacconi A., Martini P.T., Andreis I.B. (1998). A new volumetric CT machine for dental imaging based on the cone-beam technique: Preliminary results. Eur. Radiol..

[41] Hussain A., Packota G., Major P., Flores-Mir C. (2008). Role of different imaging modalities in assessment of temporomandibular joint erosions and osteophytes: A systematic review. Dentomaxillofac. Radiol..

[42] Horner K. (1992). Quality assurance: 1. Reject analysis, operator technique and the X-ray set. Dent. Update.

[43] White S., Pharaoh M. (2004). Oral Radiology Principles and Interpretation.

[44] Spin-Neto R., Wenzel A. (2016). Patient movement and motion artefacts in cone beam computed tomography of the dentomaxillofacial region: A systematic literature review. Oral Surg. Oral Med. Oral Pathol. Oral Radiol..

[45] Bontempi M., Bettuzzi M., Casali F., Pasini A., Rossi A., Ariu M. (2008). Relevance of head motion in dental cone-beam CT scanner images depending on patient positioning. Int. J. Comput. Assist. Radiol. Surg..

[46] Yildizer Keris E., Demirel O., Ozdede M. (2020). Evaluation of motion artifacts in cone-beam computed tomography with three different patient positioning. Oral Radiol..

[47] Spin-Neto R., Hauge Matzen L., Hermann L., Fuglsig J.M.d.C.e.S., Wenzel A. (2021). Head motion and perception of discomfort by young children during simulated CBCT examinations. Dentomaxillofac. Radiol..

